# A Ready-to-Use Metal-Supported Bilayer Lipid Membrane Biosensor for the Detection of Phenol in Water

**DOI:** 10.3390/membranes11110871

**Published:** 2021-11-12

**Authors:** Christina G. Siontorou, Konstantinos N. Georgopoulos

**Affiliations:** Laboratory of Simulation of Industrial Processes, Department of Industrial Management and Technology, University of Piraeus, 80 Karaoli and Dimitriou Str., 18534 Piraeus, Greece; kosnosgeo@unipi.gr

**Keywords:** BLM, metal-supported, biosensor, self-assembly, tyrosinase, water monitoring, phenol

## Abstract

This work presents a novel metal-supported bilayer lipid membrane (BLM) biosensor built on tyrosinase to quantitate phenol. The detection strategy is based on the enzyme–analyte initial association and not the commonly adopted monitoring of the redox cascade reactions; such an approach has not been proposed in the literature to date and offers many advantages for environmental monitoring with regard to sensitivity, selectivity, reliability and assay simplicity. The phenol sensor developed herein showed good analytical and operational characteristics: the detection limit (signal-to-noise ratio = 3) was 1.24 pg/mL and the sensitivity was 33.45 nA per pg/mL phenol concentration. The shelf life of the tyrosinase sensor was 12 h and the lifetime (in consecutive assays) was 8 h. The sensor was reversible with bathing at pH 8.5 and could be used for eight assay runs in consecutive assays. The validation in real water samples showed that the sensor could reliably detect 2.5 ppb phenol in tap and river water and 6.1 ppb phenol in lake water, without sample pretreatment. The prospects and applicability of the proposed biosensor and the underlying technology are also discussed.

## 1. Introduction

Phenol and phenol derivatives have a well-established toxicity profile, while their low biodegradability, their high bioaccumulation potential and their reactivity raise major environmental concerns. Sources include municipal waste, industrial effluents and agricultural runoffs, although many natural processes play a significant role in the production, accumulation and mobility of phenols in soil and sediments [1]. High toxicity has been demonstrated for more than 165 phenolic compounds [2]; the water purity standard is normally set between 1 and 10 ng/mL, depending on the drinking-water source. According to EU Council Directive 98/83/EC, the maximum allowable limit for phenol in drinking water is 0.5 ng/mL. Consequently, the rapid and reliable detection of low (or trace) phenol concentrations in water, sediment and soil is crucial for environmental assessment and management.

Phenol detection in water has been proposed using a variety of methods and techniques (see, e.g., [3]). Chromatographic techniques are commonly used for the simultaneous determination of a number of phenols in environmental samples. Liquid chromatography, combined with UV [4] or electrochemistry [5], presented detection limits ranging between 0.13–1.83 μg/mL and 0.017–0.126 μg/mL, respectively. Gas chromatographic methods reported slightly lower detectability; combined with mass spectrometry [6], a detectability of 0.1–0.5 μg/mL has been achieved for seven phenols in river sediment, whereas flame ionization [7] allowed the reliable detection and discrimination of eleven phenols at 0.005–0.120 μg/mL detection limits.

Alternative technologies have been developed within the scope of simplifying the measurement process, reducing the cost of instrumentation, shortening the time of analysis, decreasing the detection limits and supporting downsizing and portability. Nanosensing approaches expand the methods of analysis. For example, a binary metal oxide microcube-based glass carbon electrode has been recently proposed for the selective capturing of p-nitrophenol [8]; the sensor exhibits a large dynamic range (1.0–0.01 mM) and a 0.13 pM detection limit at a sensitivity of 7.12 µA/µM cm^2^. Further, Ag_2_O/Sb_2_O_3_ nanoparticles deposited on a glassy carbon electrode responded linearly to 3-methoxyphenol over a range of 0.09 nM to 0.09 mM [9]. Chromium (III) oxide nanomaterials-decorated carbon nanotubes responded to 4-methoxyphenol; the linear range was 0.01–0.1 mM, the selectivity was calculated as 1.4768 μA/mM cm^2^ and the limit of detection was 0.06428 ± 0.0002 nM [10]. The use of Ce-doped ZnO nanostructures exhibited a detectability of 11.5 ± 0.2 pM [11].

Immunoassays have been generally suggested as more suitable for the development of reliable field devices. Generally, they exhibit sufficiently low detection limits but extensive analysis times. For example, an ELISA assay was developed, using quantum dots conjugated with bisphenol A antibodies [12]; the limit of detection reported was 13.1 ng/mL and the recoveries of bisphenol A from water samples ranged between 85.92% and 109.62%. An indirect competitive immunoassay has also been suggested using gold nanoparticles on glassy carbon electrodes; discrimination between three phenol species has been demonstrated, and the detection limit was 0.25 ng/mL [13]. Microchip capillary electrophoresis was tested for the detection of phenol, dichlorophenol and trichlorophenol in landfill leachate [14]; phenol could be determined at 37.6 ng/mL and recoveries ranged between 85% and 103%. Electrochemical sensing with CdSe/ZnS core/shell type quantum dots on a glass carbon electrode exhibited good reproducibility, quick response and reliability for chlorophenol detection in environmental samples [15]; the detection limit reported was 3.355 pg/mL. Silver-doped neodymium oxide aggregated nanoparticles were reported to show a detectability of 0.06 pg/mL [16]; similar detectability has been reported with neodymium oxide co-doped zinc oxide nanostructures [17].

**Figure 1:**
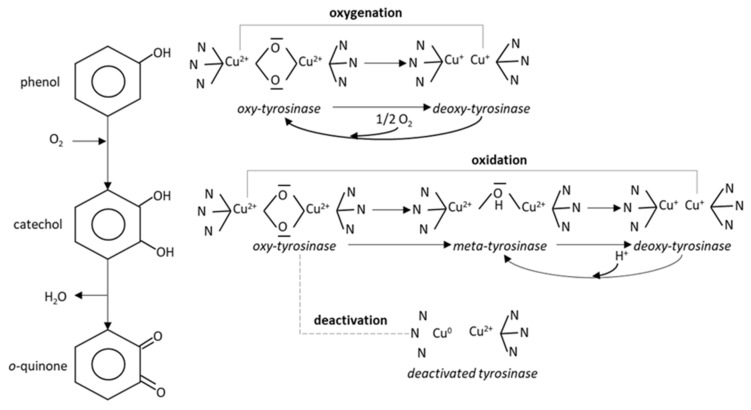
Tyrosinase catalyzed oxidation of phenol: phenol is hydroxylayted to catechol, which is dehydrogenated to o-quinone (left) or processed via the mono-oxygenase pathway (top right). Catechol follows the oxidase pathway (middle right); if it enters the mono-oxygenase pathway, the enzyme is deactivated (bottom right).

Biosensing has also been suggested for quick response, simplified assays and cost efficiency. Electrochemical formats have been mostly published using horseradish peroxidase [18], laccases [19] or tyrosinases [20]. Peroxidase is reduced by phenols and then oxidized by hydrogen peroxide [18]; phenols act as an electron mediator in this system. Peroxidase, however, is not adequately selective for phenol. Peroxidase-based sensing can serve a wide range of direct or mediated electron transfer strategies for a variety of target analytes via bio-oxidation and bio-hydroxylation (e.g., glucose, ethanol, phenylalanine, polyaromatic hydrocarbons, etc.) [21]; thus, selectivity towards phenol may be largely compromised due to interference. Phenol oxidases (i.e., laccases and tyrosinases) have a slightly different mode of action: the enzyme molecules are oxidized by oxygen and then they are reduced by phenols. The immobilization of laccase on the transducer surface has been proven to be quite challenging, impacting adversely on enzyme activity and sensor performance [22]. Tyrosinase is more frequently studied and easier to handle, but its catalytic mechanism is complicated: the enzyme has two different catalytic activities at the same binding site, and it may exist in three readily interchangeable forms (meta, oxy and deoxy), depending on the presence of oxygen and the copper oxidation state [23,24] (Figure 1). The oxidation of phenol produces quinone that can be electrochemically reduced back to catechol at a moderate potential. As catechol recycling amplifies the signal, amperometry-based biosensing is a simple transduction approach to phenol detection [25,26,27]. Phenol may be oxidized to o-quinone, even without the formation of the intermediary catechol (mono-oxygenase pathway). Catechol may be oxidized to o-quinones through the oxidase pathway, but the deoxy to meta interchange may also require redox exchange with other metals. Catecholic substrates may sometimes enter the mono-oxygenase pathway (i.e., be processed similarly to phenols) leading to copper reduction and enzyme deactivation.

The limits of detection reported for tyrosinase amperometric biosensors range between 1 and 10 ng/mL [25,27]; liposome-based sensing decreased detectability to 8.5 pg/mL [26]. However, the interchangeable enzyme forms render the mechanism of signal generation somewhat unreliable, affecting sensor reproducibility [26]. Further, the amplification of the redox signal and the manipulation of the enzyme on the transducer surface have been proven to be challenging [27]. Problems with enzyme leaching [25] and enzyme deactivation due to the produced catechols [27] have also been reported, necessitating the use of narrow linear ranges of detection.

Various materials have been employed in an effort to enhance sensitivity and stability in tyrosinase sensors. An optimized format has been developed on complex multilayer constructs of graphene–Au nanoparticle platforms with chitosan-bound tyrosinase [28]; the detection limit was 4.67 ng/mL, and the lifetime of the sensor was one month. Other approaches involved the entrapment of the enzyme into hybrid assemblies of polyaniline, polyacrylonitrile or nanostructured graphene, which increased stability at the expense of the detection limit [29]. Dispersed carbon nanotubes have been used to prepare a tyrosinase screen-printed electrode with high sensitivity towards phenol at a 3.25 ng/mL detection limit [30]. Tyrosinase immobilized on nanocrystalline cellulose quantum dot nanocomposites showed good linearity and a detection limit of 7.7 ng/mL [31]. However, the monitoring of redox reactivity is prone to interference from the many electroactive species normally expected to be present in environmental samples.

Lipid-membrane-based biosensors are not suitable for monitoring redox reactions and thus are not used in electron transfer sensing modes. Lipid membranes, especially bilayers (BLMs), have been proven to be excellent hosts for biorecognition moieties and extremely sensitive for monitoring association–dissociation events and aggregation phenomena on their surface [32]. Protein molecules can be simply physisorbed on the membrane surface retaining a favorable 3D conformational rotation [33]; a lipid layered construct spontaneously guides the orientation and the thermodynamic stability of biorecognition moieties, prevents loss of biological function and integrity and favors biomolecular interactions. Avoiding the cumbersome and multi-step schemes to involve crosslinkers and stabilizers, physisorption is an easy, one-step and straightforward means for constructing a biorecognition surface. The process is highly effective and reproducible but only marginally controllable and non-amenable to precision engineering [32].

The reaction between the protein on the membrane surface and an analyte at the membrane–electrolyte interface changes either the protein or the interface; this change is unavoidably transferred onto the membrane surface, impacting the surface charge density, the dipolar potential, the molecular packing or the membrane fluidity [32]. The result is a transient or permanent perturbation of the lipid continuity that modifies the transmembrane ion flux, manifested as an ion current signal. Further, the aggregation of small molecules on the membrane surface alters the membrane–electrolyte interface and/or the elasticity of the membrane, inducing changes in the transmembrane ion current [34].

In effect, the lipid bilayer serves both as an immobilization matrix for the bio-element and as a signal transduction and amplification system. Discriminatory capabilities have been also demonstrated in suspended bilayers (i.e., bilayers separating two aqueous interfaces) based on the differences of the partition coefficients of the analytes [35]. Although remarkably responsive, suspended bilayers are extremely fragile and are currently used for electrophysiology modeling [36]. To enhance stability for sensor applications, many construction methods have been proposed, mostly involving the support of the bilayer on a surface of some kind (metal, polymer, gel, etc.); generally, self-assembled bilayers on metal supports produce rugged systems but the discriminatory capabilities are compromised [32].

The interaction of phenolics with the lipid bilayer has been recently studied using a molecular dynamic simulation approach [37]; propofol, thymol, chlorothymol, carvacrol and eugenol interact with the membrane primarily via the γ-aminobutyric acid receptors. However, nonspecific interactions with the surrounding lipids contribute to the molecular event. In addition, the potency of plant phenols in helping carotenoids to stabilize the membrane against oxidative destruction has been investigated using electrically formed giant unilamellar vesicles [38]. In addition, a study of the partitioning of hydrophobic phenolic compounds in dipalmitoyl phosphatidylcholine vesicles showed that the localization and impacts differ; although the phenolics that are localized towards the center of the membrane (e.g., quercetin) adversely affected the colloidal stability of the liposomes, their partitioning near the phosphate head groups (e.g, garcinol, raloxifene and bisphenol A) provided a stabilizing effect [39].

The self-assembly of BLMs on metal supports was first introduced in 1990 by Tien and Salamon [40]. Since then, these constructs have been proven to be excellent models for studying surface chemistry, especially association–dissociation events, and robust platforms for sensor development [41]. Compared to other approaches for BLM formation or for guiding the self-assembly process, the methodology used is simple, straightforward, reproducible and reliable; supported lipid membrane technology can be easily interfaced with array-based systems by photolithographic patterning, spatial addressing, microcontact printing and microfluidic patterning [41]. Owing to their operational stability, metal-supported platforms are expected to provide valuable tools for the development of early-warning field sensors [32]. In addition, many applications have been described in the literature for drug discovery, disease management and diagnosis [42,43].

However, the sensitivity and versatility of the freely suspended BLMs cannot be reproduced on a metal surface [32]. Although the self-assembly process traps an ultra-small quantity of electrolyte between the metal surface and the lipid monolayer, the bilayer faces uneven stresses between its two interfaces (the metal and the bulk electrolyte). Thus, any change in the membrane structure is rapidly expressed as changes in the elasticity modulus [34]. As the electrolyte reservoir on one side of the membrane is not adequate to drive ion exchange, the analyte–bio-element interaction is manifested as permanent ion current increases. The detection range is largely dependent upon the biorecognition events: the more analyses performed on the same sensor, the more the current increases; practically, detection ends when ca. 650 nA is reached, since larger currents may indicate membrane destabilization and not analytical signals.

The characterization of the structure, orientation, molecular arrangement and performance of metal-supported BLM constructs has been previously studied. Extensive physicochemical characterization and gramicidin testing [44,45] revealed that the self-assembly process leads to the apparent formation of a functional bilayer structure. A model for the structure of the BLM supported on stainless steel wire (0.25 mm and 0.30 mm in diameter) was previously proposed based on the measurement of specific capacitance; the results indicated that the surface of the metal is covered with bilayers interrupted by multilayers, monolayers and metal (which are not involved significantly in the response of the sensor) [46]. Electrostriction studies [34] showed that as the edge effect from the metal surface is smaller with silver electrodes than with other metals, the capacitance of the supported BLM assumes values closer to the freely suspended formats. Transmission electron microscopy studies [47] showed thermotropic behavior similar to freely suspended BLMs, although the molecular packing appears to be more packed at lower temperatures.

In the present study, the association between tyrosinase and phenol, particularly the tyrosinase–phenol primary interaction (i.e., the binding of phenol to oxy-tyrosinase), was monitored using a metal-supported BLM platform. To the best of our knowledge, such an approach has not been proposed in the literature to date. The sensor developed showed good analytical and operational characteristics for phenol detection and was validated with real water samples (tap, river and lake). The prospects and applicability of the proposed biosensor and the underlying technology are also discussed.

## 2. Materials and Methods

### 2.1. Materials

Lipid membranes have been formed from egg phosphatidyl choline (egg PC Type XVI-E) purchased from Sigma-Aldrich (St. Louis, MO, USA) and stored at −20 °C; lipid solutions (2.5 mg/mL in 80% *v*/*v* n-hexane and 20% *v*/*v* absolute ethanol) could be kept refrigerated (0–4 °C) for up to 2 months. Teflon-coated silver wires (diameters of 0.1, 0.25, 0.5 and 1.0 mm) were also purchased from Sigma-Aldrich. Tyrosinase from mushroom (polyphenol oxidase, E.C.1.14.18.1, 5370 U/mg, Sigma-Aldrich) was diluted/reconstituted in buffer to obtain stock solutions; when not in use, stock solutions were kept refrigerated (4–8 °C). High-purity phenol (>99.5%) was obtained from Fluka (Buchs, Switzerland). Catechol was obtained from Sigma-Aldrich. All other chemicals were reagent grade and obtained from Sigma-Aldrich.

Electrolyte solutions were prepared from KCl and HEPES (N-2-hydroxyethyl-piperazine-N’-2-ethanosulfonic acid). The water used was either distilled through a filtering system or purified by passage through a Milli-Q cartridge filtering system (Milli-Q, Millipore, El Paso, TX, USA).

### 2.2. Apparatus

**Figure 2:**
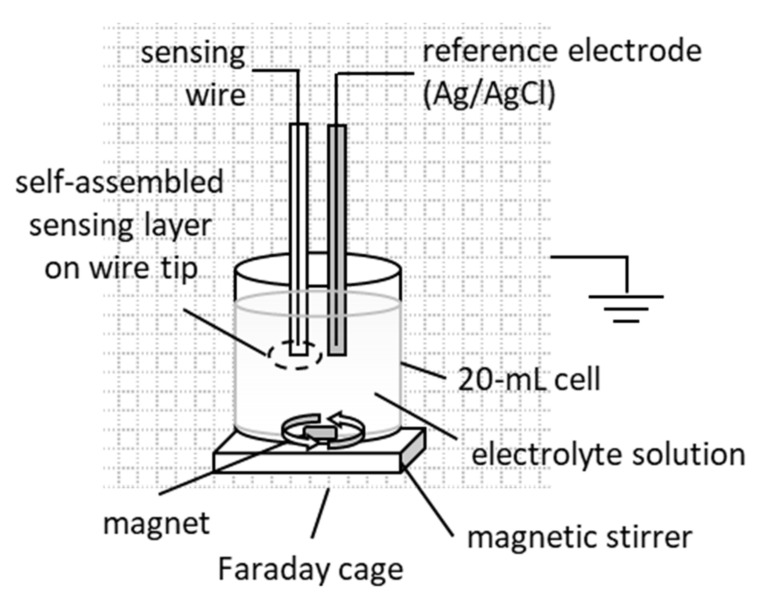
Overview of the electrochemical set-up for metal-supported biosensors. The applied potential at the sensing electrode is positive relative to ground. Not drawn to scale.

The electrochemical set-up used for the metal-supported biosensors described herein is presented in Figure 2. A two-electrode configuration system was assembled, i.e., a sensing wire hosting the lipid membrane/bio-element complex on its tip and a reference Ag/AgCl electrode, both immersed in electrolyte. The reference wire was prepared as follows: the coating was removed in a small area around the tip of the wire, which was lightly sanded, immersed in chloride solution for a short time and rinsed off with distilled water.

A power source (SourceMeter^®^, Keithley 2400, OH, USA) supplied a 25 mV DC potential, and the current between the sensing and the reference electrodes was measured with an electrometer (System Electrometer, Keithley 6514, OH, USA) that also served as a current-to-voltage converter. The electrochemical cell and electronic equipment were placed in a grounded Faraday cage (homemade). LabVIEW (National Instruments Co., Austin, TX, USA) properly customized for small currents was used to store and process signal data. All experiments were performed at 25 ± 1 °C. Statistical analysis was performed using GraphPad Prism version 8.0.0 for Windows (GraphPad Software, San Diego, CA, USA).

### 2.3. Sensor Assembly

**Figure 3:**
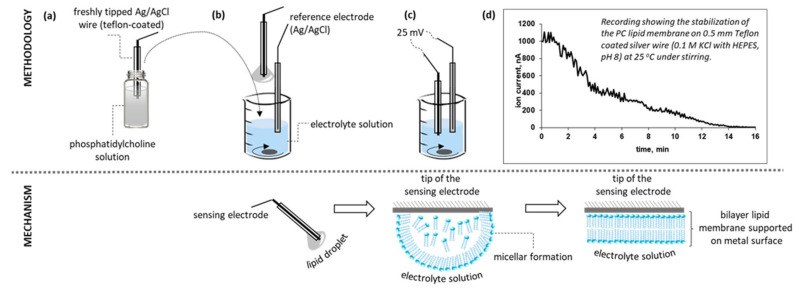
Schematic overview of the construction of the metal-supported bilayer lipid membrane. The stages of the self-assembly process are shown on the lower panels. (**a**) Immersion of the sensing wire into the lipid solution. (**b**) Transfer of wire to the electrolyte solution under stirring. (**c**) Initiation of the self-assembly process. (**d**) Monitoring of the self-assembly process. Please note that the representation of the self-assembly process is only figurative. Not drawn to scale.

Metal-supported phosphatidyl choline (PC) lipid membranes were constructed from the stock lipid solution of 2.5 mg/mL (in 10 mL of 80% *v*/*v* n-hexane and 20% *v*/*v* absolute ethanol), as previously described [44,45,48]. Briefly, the sensing wire was tipped just before its immersion in the lipid solution; the wire was subsequently immersed in the electrolyte solution and a 25 mV voltage was applied between the electrodes (Figure 3).

The lipids spontaneously organized into a micellar formation, orienting their polar heads tangentially to the metal surface in order to securely place their hydrophobic tails inwards. The micellar formation thinned due to the electrostatic interactions into multilayer formats that finally degraded into a bilayer.

The lipid self-assembly process could be monitored through current stabilization; a steady background current could be reached within ca. 10–15 min on 0.5 mm and 1.0 mm silver wires. Slightly longer stabilization times (ca. 20 min) were observed with thinner wires. The membrane was not treated further. Following the stabilization of the background ion current, 10 μL of the enzyme stock solution (5 mg/mL) was injected into the bulk electrolyte under mild stirring. Physisorption being a slow process, a stable background current was reached within 6–8 min.

### 2.4. Construction of the Tyrosinase Metal-Supported Lipid Membrane Sensor

The metal-supported lipid membranes were constructed in this study from PC. The optimal concentration of the lipid solution was investigated in the range of 1–3 mg/mL (as the concentration in bulk). The use of a 2.5 mg/mL lipid solution had a 100% success rate in constructing the lipid platforms, with stabilization times achieved within 10–15 min for a 0.5 mm diameter sensing wire; more dilute lipid solutions gave a success rate of 60%, whereas higher concentrations resulted in prolonged stabilization times.

The use of sensing wires with a diameter of 0.5 mm produced more rugged platforms (280 mV breakdown voltage) and 30% shorter membrane stabilization times than the 0.1 mm wires; the background ion current was 15.8 ± 4.2 nA, somewhat higher than that observed with the 0.1 mm wires but adequate for sensing. When a 1.0 mm diameter wire was used, the background ion current increased to 60 nA, limiting the analytical range considerably.

Once formed and stabilized, the sensing wire–lipid membrane conjugate remained functional (i.e., electrode drift <5%) within the electrolyte solution for 30 h at ambient temperature. Outside the electrolyte solution, i.e., in air, stability was demonstrated for ca. 10 min; upon re-immersion, the membrane regained its functionality after ca. 2 min. The metal-supported membrane was consistently extremely stable upon experimentation for >8 h; pH changes on the membrane surface, within the pH range studied herein, did not induce membrane instabilities, at least to the extent that they would adversely affect the integrity of the membrane [49]. Similar behaviour was also observed in previously developed metal-supported lipid membrane sensors [44,45,48]. In addition, many studies on acid–base equilibria on a BLM surface concluded that pH shifting, especially at pH values >4.0, increased the interfacial tension of PC membranes and the value of their capacitance, leading to the formation of disturbances in membrane symmetry [50,51]. This effect could be used as a very successful signal amplification mechanism. At pH values >7.0, the pH increase resulted in an increase in the surface charge density of the bilayer that enhanced further the responsivity of the membrane [52,53].

### 2.5. Treatment of Environmental Samples

Matrix effects for aqueous environmental samples were simulated using solutions of varying compositions between anions (carbonates, nitrates, chloride, sulfates, sulfides, cyanides and phosphates), cations (calcium, magnesium and ammonium ions), glucose, uric acid and amino acids (alanine, aspartic acid, glutamine, glycine, phenylalanine, tryptophan and tyrosine).

Real water samples were collected, kept refrigerated (4–8 °C) and used without further treatment. Lake samples were collected in mid-March from Koumoundourou Lake (Attiki, Greece). The salinity of the lake was generally very high (1.2% and 14.7%), varying significantly due to irregular freshwater inflow from submerged springs. The biochemical oxygen demand (BOD) was low and the chemical oxygen demand (COD) values fluctuated between low and moderate [54]; the lake had high nitrogen concentrations, especially as N-NO_3_ (1237–1394 mg/L) and extremely low phenol levels (<0.5 ng/mL). The average pH of the samples taken was 6.5 ± 0.5.

River samples were collected in early April from a mixed spring and rainwater stream in a small mountainous settlement of Northern Greece (Ioannina Prefecture: 39°41′29.040″ N; 21°2′17.880″ E; 750 m altitude; 982 mm mean annual precipitation). The average pH of the samples taken was 7.7 ± 0.3. Tap water, collected in late February from the public water supply network, had a pH of 7.8 ± 0.2.

Airborne phenol may be a source of phenol contamination in samples and sample extracts. In order to avoid the chemical contamination of the target phenol analyte, all experiments (including sensor development, sample storage and assay) were performed in an area where phenol is not used for other laboratory operations.

## 3. Results and Discussions

### 3.1. Biosensor Functioning

**Figure 4:**
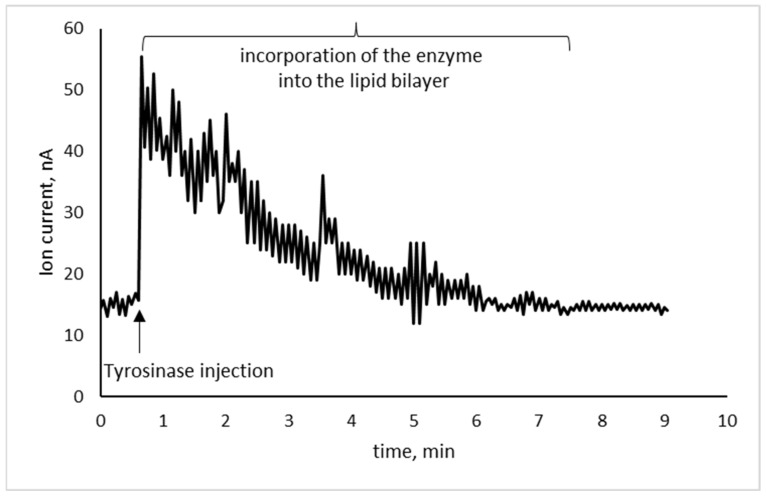
Recording showing the incorporation of the enzyme into the metal-supported BLM under stirring (PC membrane; 2.5 μg/mL tyrosinase as concentration in bulk; 0.1M KCl buffered with HEPES; 0.5 mm diameter Teflon-coated silver wire; 25 °C).

Enzyme integration was investigated with lipid membranes self-assembled on a 0.5 mm sensing wire, using 0.1M KCl electrolyte solution buffered with HEPES. Following the addition of the enzyme, at a concentration of 2.5 μg/mL of tyrosinase in the bulk, the ion current stabilized within 6–8 min, giving a background current of 15 ± 2.5 nA, (*n* = 12), comparable to that obtained without the enzyme (Figure 4); it is thereby indicated that the enzyme–lipid interactions are not predominantly electrostatic and the immobilization of the enzyme on the membrane surface does not induce poration [55].

Alternatively, the enzyme could be added to the lipid mixture prior to membrane formation, so that immobilization could occur during the self-assembly process. As observed, current stabilization required >45 min, the background current was much higher (20–25 nA) and sensor reproducibility (as indicated by the sensor’s response towards a given phenol concentration) decreased by 42%.

### 3.2. Phenol Detection

In the absence of the enzyme, the lipid membrane exhibited no discernible selectivity towards phenol at pH 8, even at very high concentrations; no changes were observed in the background ion current or the noise levels. At pH values between 5.5 and 7.5, 50 pg/mL of phenol (i.e., a concentration 3 times higher than the upper analyte concentration level) increased noise levels slightly; this increase became more prominent as the pH decreased, reaching a maximum value of 21% at pH 5.5. These results are consistent with earlier studies on the partitioning of phenol in phospholipid vesicles showing that only the uncharged phenols influence the fluidity of the lipid membrane, but the effect is pH-dependent [56]: at pH 7, limited adsorption was demonstrated but at higher pH values, phenol was insoluble in the lipid and at pH values < 5, partitioning into the hydrophobic chains was concentration-dependent. Catechol, a possible intermediate in the tyrosinase–phenol interaction has been found to interact weakly with the outer leaflet of a model bilayer through limited headgroup partitioning at pH 7.4 [57]. No response was recorded in the metal-supported lipid membrane sensor presented herein from the addition of catechol concentrations up to 1M at pH 8.

The monitoring of the enzyme–analyte interaction in the bulk (i.e., with unbound enzyme) produced no discernible membrane response. At pH values < 7, small transient ion currents were recorded, possibly due to small-scale electrostatic changes at the surface of the membrane, without analytical use.

The tyrosinase membrane sensor responded to phenol additions by permanent ion current increases, the magnitude of which was linearly related to the concentration of the analyte in the bulk. The response time obtained was 10 ± 0.75 s (*n* = 31), suggesting rapid alterations at the surface of the membrane. This response probably demonstrated initial enzyme–analyte interactions but the response time was too short to accommodate any downstream interactions. In any case, the sensor was not built to monitor reduction currents and, further, after the appearance of the ion current increase corresponding to the addition of the analyte, no signal was recorded for 15 min.

The signals obtained at increasing phenol concentrations had such large magnitudes that they could not be attributed simply to surface membrane alterations due to the enzyme–phenol interactions; knowing that the complexation of the enzyme with phenol changes the state of the enzyme [24], concurrent changes in the membrane packing and fluidity seem possible. In addition, the response of the sensor towards phenol was the same, regardless of whether the analyte was introduced by stepwise additions or as single injection. For example, when the phenol bulk concentration reached 3.72 pg/mL with stepwise additions, the mean difference in the ion current (ΔI) was 128.7 ± 10.1 nA (*n* = 10); single injections of 3.72 pg/mL phenol in the bulk, produced signals of 130.7 ± 10.9 nA (*n* = 10). Similar results were obtained for 13.64 mg/mL of phenol, indicating no memory effects. Thus, the sensor developed exhibits no statistically significant carryover effects and can be used for multiple analyses.

The effect of enzyme loading on signal magnitude was studied in the range 1.25–10 μg/mL (concentration in bulk). The optimal bulk tyrosinase concentration was found to be 2.5 μg/mL; lower levels did not provide adequate sensitivity for detection (as shown by the 33% reduction in the response of the sensor to 3.72 pg/mL of phenol in the bulk), while higher levels caused membrane destabilization.

The effect of pH was also investigated in an effort to determine the optimal conditions for maximum signal generation. The sensitivity of the sensor, calculated from the slope of the calibration curve in the linear range and defined as the proportion of change in the response of the system when the concentration of the analyte increases by a unit degree, is largely dependent upon transduction [58]. Furthermore, the detection limit, characterized as the lowest analyte concentration that can be reliably determined, is calculated as the analyte concentration that triggers a signal with a magnitude three times that of the noise; thus, the higher the ionic currents, the lower the detectability and the lower the sample volumes required to achieve that detection limit [59].

**Figure 5:**
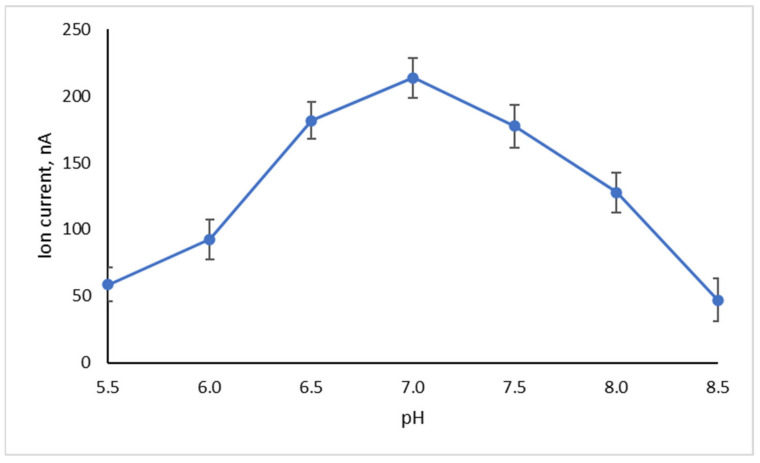
The effect of pH on the response of a metal-supported PC membrane incorporating 2.5 μg/mL tyrosinase (0.1M KCl buffered with HEPES; 0.5 mm diameter Teflon-coated silver wire; 25 °C) towards 3.72 pg/mL phenol (as concentration in bulk). Error bars denote standard deviation (*n* = 5). For more details, see text.

Other tyrosinase biosensors have been reported with optimal pH values between 6 and 7 [25,26,27]; at lower values, the produced catechol may replace phenol at the enzyme binding sites leading to enzyme deactivation [24], while higher values do not favor the mono-oxygenase pathway [27]. Using a phenol bulk concentration of 3.72 pg/mL, the highest signal, i.e., 213.8 nA, was achieved at pH 7.0 (Figure 5). However, the destabilization of the sensor became apparent after two consecutive phenol injections, manifested as an increase in the background ion current to 420 nA, with multiple transient signals of high magnitude that prohibited any further use of the sensor. This effect could possibly be attributed to membrane fluidity alterations due to enzyme state shifting and/or catechol interference (provided that catechols were indeed produced in situ).

At pH values < 7, increased noise levels were observed; at pH 5, noise levels became excessively high, with transients up to 100 nA. While phenol partitioning cannot be excluded [60], enzyme deactivation or desorption from the bilayer is also possible [61]. At pH values > 8.5, the response of the sensor towards phenol dropped to 5.61% of its value at pH 7. At pH 8.0, the response of the sensor decreased by 40%, but the system remained stable and functional after ten consecutive injections, where it reached the maximum allowable current level. At this pH, no interference is expected by non-specific interactions between the analyte or the oxidation by-products.

**Figure 6:**
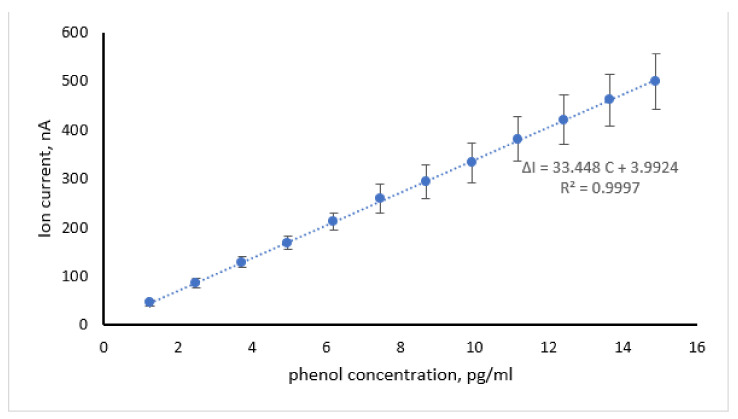
Calibration graph for phenol detection using metal-supported PC membranes incorporating tyrosinase (2.5 μg/mL, concentration in bulk). Experimental conditions: pH 8.0; 0.5 mm Teflon coated Ag wire; 0.1 KCl solution with HEPES; 25 °C. Error bars denote standard deviation (*n* = 31). For more details, see text.

The calibration graph for phenol detection under the optimized conditions is shown in Figure 6. Calibration was performed by stepwise additions of phenol standard solution under stirring, in order to demonstrate the reliability of the sensor (mainly the absence of carryover or memory effects). The coefficient of determination (R^2^) was 0.9997 (*n* = 31) and the reproducibility of the response was estimated at ±8–12% for within-day analyses (as the relative standard error, *n* = 31, 5.95% confidence limit).

Between days and analysts, precision was estimated over five days and three analysts for three phenol concentrations (Table 1); two-way ANOVA results are also presented (Table 2). A fresh sensor was used for each analyst and day (i.e., each analyst used stepwise additions of phenol to yield the response to the three concentrations tested), while the estimation of phenol concentration in the bulk was based on the calibration graph of Figure 6 (i.e., the sensor was not re-calibrated).

The results indicate the reproducibility of the response, with no positive or negative trend, indicative of standard errors and carryover effects. Standard deviation values were within the range of the calibration curve; the error of measurement was <5% in all cases, whereas a day or analyst significant effect was not observed, as shown by the corresponding *p*-values in Table 2 (ranging between 0.077 and 0.8587). The precision of the measurements was found to be 4.82%, 4.42% and 3.57% for the low, medium and high phenol concentrations, respectively, whereas the accuracy was estimated as 2.53%.

The linear range was 1.24–15 pg/mL, with a detection limit (as S/N = 3) of 1.24 ± 0.6 pg/mL (*n* = 7) and a sensitivity of 33.45 nA per pg/mL phenol concentration. Some drift of the ion current with time was noticed, especially at high phenol concentrations and prolonged use; however, the maximum value observed was 1 nA/min, increasing after 12 h of storage in electrolyte at room temperature. The lifetime (in consecutive assays) was 8 h. A comparison of the sensor developed herein with other phenol detection methodologies is provided in Table 3. The detection limit achieved herein was lower than that reported by other tyrosinase biosensor systems [25,26,27] and higher sensitivity was demonstrated, comparable to chromatographic detection [4,5,6,7], immunoassay formats [12], electrophoretic devices [14] or ZnO nanostructures [11].

### 3.3. Sensor Reversibility

Earlier metal-supported platforms demonstrated the response of protein [44] and DNA [45] lipid membrane sensors towards decreasing analyte concentrations, leading to sensor reversibility, i.e., the restoration of low ion currents allowing the reuse of the same sensor for another experimental run. Although the reversibility mechanism was only investigated electrochemically, decreasing the analyte concentration in the bulk yielded a concentration gradient between the membrane surface and the bulk electrolyte. This gradient was adequate to pull away analyte molecules from the electrolyte–membrane interface. The decomplexation of the analyte from DNA has not been proven [45]; furthermore, limited DNA desorption was possible, as evidenced by the lag time required to re-establish a stable system. However, the complete extraction of DNA from the membrane was not feasible without destroying the sensor.

The capability of the sensor developed herein to monitor decreasing phenol concentrations was investigated as a means of sensor regeneration. The phenol-rich electrolyte was gradually removed from the electrochemical cell and replaced with phenol-free electrolyte; the procedure stopped when the ion current dropped to acceptably low levels. Compared to calibration (Figure 6), the response of the sensor towards decreasing phenol levels was rather elevated while the sensitivity was slightly decreased, possibly indicating memory and carryover effects. Washing the sensing electrode with strongly acidic or alkaline solution has been suggested in the literature [61]; as lipids are oxidized at pH values < 4.5, only the alkaline treatment was considered herein.

The study of pH (Figure 5) showed that the response of the sensor towards phenol was minimal at pH 8.5. Filling the electrochemical cell with 15 pg/mL phenol (the highest analyte concentration tested) and after receiving the corresponding signal and allowing the system to stabilize, the sensing wire was removed from the electrochemical cell and immersed in pH 8.5 buffer (0.1M KCl with HEPES) for 10 min. When the sensing wire was transferred into phenol-free and enzyme-free electrolyte (pH 8.0), the ion current stabilized to 28 ± 1.5 nA (*n* = 12), 20% higher than the background ion current, within 5–6 min; the sensitivity towards phenol was not affected (within analytical error), clearly indicating insignificant or no enzyme desorption. Shorter washing times settled the ion current at higher values.

The number of repeated assays of high-phenol samples that could be achieved, including analysis, washing and re-stabilization, was eight, without observing any statistically significant reduction in signal magnitudes. The number of samples that could be assayed with the same sensor depended on phenol concentration in the sample; at 1.24 pg/mL phenol, the number of assay repetitions that could be performed increased to 15.

### 3.4. Sensor Validation

Possible interference from a number of anions (carbonates, nitrates, phosphates, chloride, sulfates and sulfides) and cations (ammonium, calcium and magnesium ions) was studied herein with simulated water samples, at varying compositions and concentrations (Table 4). The sensor exhibited high tolerance to interfering ions during simulation; the results showed a determinant error < 5% for bulk electrolyte concentrations up to the high mM range.

Phenol-spiked tap-, river- and lake-water samples were used for validation studies. Spiked samples were freshly prepared from the phenol stock solution and analyzed immediately. For tap and river water, 10 mL samples were spiked with phenol to reach a concentration of 9.4 ng/mL (near the upper allowable level for drinking water). For lake water, 5 mL samples were spiked with phenol to reach a concentration of 18.8 ng/mL; a lower concentration than the allowable phenol limit was chosen in order to elucidate matrix effects. A 10 μL assay volume was used. The same sensor was used to analyze all samples with a mean analysis rate of 14 samples/h (including regeneration); the calibration graph of Figure 5 was used for quantitation (i.e., the sensor was not re-calibrated), taking into account the dilution factors.

Tap and river water did not affect the sensor characteristics; lake water increased noise levels by 42.86%, necessitating the increase of the detection limit to 2.8 pg/mL. Phenol recovery in the tap-water samples ranged between 93 and 105% (Table 5), with no positive or negative trends, indicative of standard errors. For environmental samples, the acceptable range is 80–115% [62]. Considering sample dilutions, the lowest phenol concentration in tap-water samples that could be reliably detected was 2.5 ng/mL. Similar results were obtained for the river-water samples (Table 5); phenol recovery ranged between 92 and 104%. The results for tap and river water are comparable with the standard assay validation (Table 1), although the deviation observed in tap water was slightly higher. It is worth noting that the drinking water of the Attiki region is supplied by four lake reservoirs.

Table 6 presents the results from the lake-water samples; a consistent positive trend was observed possibly due to matrix effects or/and phenol already present in the sample. Previous studies of the lake reported very low phenol levels, medium to high salinity and high levels of nitrates [54]. Nevertheless, the maximum deviation was +6.6%, allowing for reliable detection of lake samples. Considering sample dilutions, the lowest phenol concentration in lake-water samples that could be reliably detected was 6.1 ng/mL.

### 3.5. Marketability and Miniaturization

In situ monitoring and control necessitates the development of fit-for-purpose field sensors with the required accuracy, the ability to be constructed and handled by untrained personnel and the ability to be produced with local resources. Metal-supported platforms are reproducible, with easy assembly, offering simple instrumentation at an affordable cost and requiring only easily acquired expertise. Further, the time required to complete analytical investigations may be considered short to medium. This is a significant advantage over other analytical techniques such as spectroscopy or chromatography, where the preparation of the system requires longer times.

A competitive advantage for niche applicability was demonstrated for the bench-scale set-up developed herein, especially in terms of its proven capabilities for direct analysis of real samples. Hence, miniaturization and design for mass production should be investigated in depth. Preliminary results suggest that the electrochemical cell can be downsized to 10 mL or 5 mL and that 1 μL sample injectors can be used. Further reduction requires thinner sensing wires but noise compensation should be addressed. Chip integration or screen printing might not be feasible given the lipid membrane self-assembly process employed; microelectromechanical technology and microfluidics might decrease the sensor to hand-held size, providing some automation of lipid membrane formation. Although much work is still required to convert the sensor developed into a field detector, on-the-go multi-analyte monitoring might feasibly be within reach.

## 4. Conclusions

This work presented a novel metal-supported lipid membrane biosensor built on tyrosinase to quantitate phenol. The detection strategy was based on the enzyme–analyte initial association and not the commonly adopted monitoring of the redox cascade reactions. Such an approach has not been proposed in the literature to date and offers many advantages for environmental monitoring with regard to sensitivity, selectivity, reliability and assay simplicity.

The detection limit achieved was lower than that reported by other tyrosinase biosensor systems [25,26,27] and higher sensitivity was demonstrated, comparable to chromatographic detection [4,5,6,7], immunoassay formats [12], electrophoretic devices [14] or ZnO nanostructures [11]. The sensor produced exhibited a high tolerance to interference and matrix effects without the need for sample pretreatment or other laborious strategies. Further, the analytical range achieved and the ultra-low detection limit (1.24 pg/mL) are quite suitable to serve the environmental norms for water quality. Higher sample concentrations can be assayed with further sample dilution.

The metal-supported lipid membrane platform is easily and reproducibly constructed by minimally trained personnel. The physical chemistry of the membrane offers a bio-element-compatible environment and a built-in signal amplification tool. Bio-element incorporation through physisorption might not be material-effective but it is definitely easy, reproducible over a large number of sensors and reliable over a long period of time. This was proven by the validation studies described herein, where a large number of operating sensors were used without re-calibration.

The shelf life of the tyrosinase sensor was 12 h and the lifetime (in consecutive assays) was 8 h. Operational functionality was also demonstrated using real environmental samples without sample pretreatment; the sensor could reliably detect 2.5 ng/mL (i.e., 2.5 ppb) of phenol in tap and river water and 6.1 ng/mL (i.e., 6.1 ppb) in lake water. Although the results from the environmental samples obtained should be verified further with a more extended study, proof of concept has been provided and the phenol sensor developed is readily applicable. Further, a competitive advantage for niche applicability has been demonstrated for the bench-scale set-up developed herein, especially in terms of its proven capabilities for direct analysis of real samples.

The short assay times (ca. 5 min) and small sample volumes (5–10 μL) are suitable for routine analysis with the inclusion of electrode washing into the assay protocol. If needed, sensor reconstruction is easy; it can be performed in advance and the membrane platform can be stored for >30 h without the bio-element. In any case, the operational lifetime for the sensor is limited to one working day and the storage stability to 12 h.

The proposed phenol detection scheme might be also suitable for the simultaneous detection of phenolic and polyphenolic compounds. Further work is required in order to clarify the selectivity of the sensor towards phenolics and its discriminatory capability. Nevertheless, the sensor design strategy presented could be promising for enhancing the development of fit-for-purpose and customized sensors.

## Figures and Tables

**Table 1:** Intermediate precision results for phenol detection with the metal-supported lipid membrane biosensor incorporating 2.5 μg/mL tyrosinase (0.1M KCl buffered with HEPES; 0.5 mm diameter Teflon-coated silver wire; pH 8.0; 25 °C).

Day	Phenol (pg/mL)	Signal 1/Analyst 1	Signal 2/Analyst 2	Signal 3/Analyst 3	Mean	SD	Estimated Concentration	Error of Measurement
1	2.48	90	88	95	91.0	3.61	2.601	+4.89%
	3.72	120	128	119	122.3	4.93	3.538	−4.89%
	6.20	202	199	210	203.7	5.69	5.970	−3.71%
2	2.48	78	85	90	84.3	6.03	2.402	−3.15%
	3.72	128	125	130	127.7	2.52	3.698	−0.60%
	6.20	200	195	220	205.0	13.23	6.010	−3.07%
3	2.48	86	89	85	86.7	2.08	2.472	−0.33%
	3.72	125	125	122	124.0	1.73	3.588	−3.55%
	6.20	225	210	218	217.7	7.51	6.388	+3.04%
4	2.48	80	94	87	87.0	7.00	2.482	+0.07%
	3.72	127	122	130	126.3	4.04	3.658	−1.68%
	6.20	225	212	198	211.7	13.50	6.209	+0.14%
5	2.48	85	90	92	89.0	3.61	2.541	+2.48%
	3.72	129	133	125	129.0	4.00	3.737	+0.47%
	6.20	215	200	199	204.7	8.96	6.000	−3.23%

**Table 2:** Two-way ANOVA results.

2.48 ng/mL	Sum of Squares	Degrees of Freedom	Mean Square	F (DFn, DFd)	*p*-Value
analyst	109.2	2	54.60	(2, 8) = 3.576	0.077
day	76.27	4	19.07	(4, 8) = 1.249	0.3644
residual	122.1	8	15.27		
Source of variation		% of total variation		*p*-value	
analyst		35.50		0.077	
day		24.79		0.3644	
3.72 ng/mL	Sum of squares	Degrees of freedom	Mean square	F (DFn, DFd)	*p*-value
analyst	4.933	2	2.467	(2, 8) = 0.1553	0.8587
day	87.73	4	21.93	(4, 8) = 1.381	0.3225
residual	127.1	8	15.88		
Source of variation		% of total variation		*p*-value	
analyst		2.245		0.8587	
day		39.93		0.3225	
6.20 ng/mL	Sum of squares	Degrees of freedom	Mean square	F (DFn, DFd)	*p*-value
analyst	261.7	2	130.9	(2, 8) = 1.324	0.3187
day	433.1	4	108.3	(4, 8) = 1.095	0.4211
residual	790.9	8	98.87		
Source of variation		% of total variation		*p*-value	
analyst		17.62		0.3187	
day		29.15		0.4211	

**Table 3:** Comparison of the tyrosinase metal-supported lipid membrane sensor developed herein with other methodologies for the detection of phenol in water samples.

Methodology	Detection Limit	Sensitivity	Refs
Liquid chromatography combined with UV	0.13–1.83 μg/mL	524–5593 mAU per μg/mL of phenol concentration	[4]
Liquid chromatography combined with electrochemistry	0.017–0.126 μg/mL	0.0167–0.2650 nA/min per mg/L of phenol concentration	[5]
Gas chromatography combined with mass spectrometry	0.1–0.5 μg/mL	not mentioned	[6]
Gas chromatography combined with flame ionization	0.005–0.120 μg/mL	not mentioned	[7]
Binary metal oxide microcube-based glass carbon electrode	0.018 pg/mL	7.12 µA/µM cm^2^	[8]
Ag_2_O/Sb_2_O_3_ nanoparticles deposited on a glassy carbon electrode	0.009 pg/mL	11.67 μA/μM cm^2^	[9]
Chromium (III) oxide nanomaterials-decorated carbon nanotubes	0.008 pg/mL	1.4768 μA/mM cm^2^	[10]
Ce-doped ZnO nanostructures	1.43 pg/mL	94.937 μA/μM cm^2^	[11]
ELISA/quantum dots conjugated with bisphenol A	13.1 ng/mL	not mentioned	[12]
Gold nanoparticles on glassy carbon immunoassay	0.25 ng/mL	not mentioned	[13]
Microchip capillary electrophoresis	37.6 ng/mL	not mentioned	[14]
CdSe/ZnS core/shell type quantum dots on glass carbon electrode	3.355 pg/mL	3.6392 µA/µM cm^2^	[15]
Silver-doped neodymium oxide aggregated nanoparticles	0.06 pg/mL	0.2215 μA/μM cm^2^	[16]
Neodymium oxide co-doped zinc oxide nanostructures	0.061 pg/mL	28.481 nA/nM cm^2^	[17]
Tyrosinase glass carbon sensor	1.29 ng/mL	0.256 mC/μM	[25]
Liposome bioreactor and chitosan nanocomposite tyrosinase sensor	1.02 ng/mL	not mentioned	[26]
Tyrosinase/redox polymer composite sensor	9.4 ng/mL	0.15 nA per µM of analyte concentration	[27]
Graphene–Au nanoparticle platforms with chitosan-bound tyrosinase	4.67 ng/mL	0.624 μA/μM	[28]
Hybrid assemblies of polyaniline, polyacrylonitrile and nanostructured graphene	24.9 ng/mL	6.46 μA/μM cm^2^	[29]
Tyrosinase screen-printed dispersed graphene electrode	3.25 ng/mL	1170 µA/mM cm^2^	[30]
Tyrosinase immobilized on nanocrystalline cellulose quantum dots nanocomposites	7.7 ng/mL	0.078 μA/μM	[31]
Metal-supported lipid membrane with incorporated tyrosinase	1.24 pg/mL	33.45 nA per pg/mL of analyte concentration	This work

**Table 4:** Results obtained from the response of the phenol sensor to simulated water samples containing possible interference species and 6.2 pg/mL phenol.

Matrix Composition	Signal Deviation% (*n* = 5)
Carbonates (32.78 mM as HCO_3_^−^)	0.9 ± 0.3
Nitrates (44.5 mM as NO_3_)	3.3 ± 0.2
Phosphates (12.8 mM as PO_4_^3−^)	1.5 ± 0.1
Chloride (34.44 mM Cl^−^)	3.2 ± 0.2
Sulfates (10 mM as SO_4_^2−^)	1.0 ± 0.3
Sulfides (10 mM as (NH_4_)_2_S)	0.5 ± 0.05
Ammonium (10 mM as (NH_4_)_2_S)	1.3 ± 0.6
Calcium (1.05 mM Ca^2+^)	1.7 ± 0.1
Magnesium (1.40 mM Mg^2+^)	0.4 ± 0.1
HCO_3_^−^/NO_3_/Cl^−^ (at max. concentrations)	4.2 ± 0.5
PO_4_^3−^/SO_4_^2−^ (at max. concentrations)	2.6 ± 0.7
Cl^−^/(NH_4_)_2_S (at max. concentrations)	4.4 ± 1.1
Ca^2+^/Mg^2+^/NO_3_ (at max. concentrations)	4.0 ± 0.6
HCO_3_^−^/Cl^−^/SO_4_^2−^/Ca^2+^/Mg^2+^ (at max. concentrations)	4.5 ± 0.4

**Table 5:** Validation results from the recovery of phenol in tap- and river-water samples containing 9.4 ng/mL phenol.

	Tap Water			River Water	
#	Phenol Detected with the Sensor (ng/mL)	% Relative Error	#	Phenol Detected with the Sensor (ng/mL)	% Relative Error
1	9.06	−3.617	1	9.39	−0.126
2	9.36	−0.426	2	9.63	+2.418
3	9.84	+4.681	3	9.81	+4.327
4	9.48	+0.851	4	9.03	−3.943
5	9.66	+2.766	5	8.73	−7.123
6	9.18	−2.340	6	9.57	+1.782
7	9.90	+5.319	7	9.33	−0.762
8	9.84	+4.681	8	9.81	+4.327
9	9.12	−2.979	9	8.85	−5.851
10	8.76	−6.809	10	9.15	−2.670

**Table 6:** Validation results from the recovery of phenol in lake-water samples containing 18.8 ng/mL phenol.

#	Phenol Detected with the Sensor (ng/mL)	% Relative Error
1	19.26	+2.447
2	18.96	+0.851
3	20.04	+6.596
4	19.56	+4.043
5	19.02	+1.144
6	19.49	+3.688
7	19.19	+2.098
8	18.90	+0.508
9	19.86	+5.638
10	19.80	+5.319

## Data Availability

Not applicable.
